# Supplementary material for: Are carbon emissions trading and green financial instruments synergistic? -Comprehensive quantitative research based on content analysis
Source: PLoS One. 2024 Mar 7;19(3):e0298601. doi: 10.1371/journal.pone.0298601 (PMC10919601; doi:10.1371/journal.pone.0298601)
Supplement: S1 Data — (ZIP) [file pone.0298601.s001.zip › Data/地级市Z_F工作报告.zip!/─┌├╔╣┼/░n═╖╩╨/└┤╫╘╣1⁄2╓┌║┼í╛┬φ┐╦╩2╛▌═°í┐_2012.docx]

各位代表：

　　现在，我代表市人民政府，向大会作工作报告，请予审议，并请市政协委员和其他列席人员提出意见。

　　一、2011年工作回顾

　　过去的一年，在自治区党委、政府和市委的领导下，在市人大、政协的监督和支持下，市政府团结依靠全市各族人民，深入贯彻落实科学发展观，大力实施“科学发展、全面转型、富民强市”战略，在大事多、任务重的情况下，比较圆满地完成了经济社会发展的主要目标任务，实现了“十二五”的良好开局。全市生产总值完成3005.4亿元，增长15.5%；固定资产投资完成2160.6亿元，增长21.5%；地方财政总收入完成295.5亿元，增长21.4%；城镇居民人均可支配收入29628元，农牧民人均纯收入10059元，分别增长14.6%和14.7%。节能减排实现预期目标，列入国家重点监控的减排工程效益显著。向全市人民承诺的20件32项好事实事全面完成。全市瞩目的三件大事，在各族人民的共同努力下获得成功。顺利承办了第十一届全国中学生运动会，首次进入国家卫生城市行列，第三次荣获全国文明城市称号，城市知名度和影响力进一步提升。

　　（一）工业结构不断优化。加快传统产业改造升级步伐，组织实施了239个重点项目。包钢重轨旧线改造、热轧无缝钢管和大安、吉宇技改升级等钢铁产业项目，包铝技改升级、东方希铝铝板带、成功铝业铝型材、常熟铝业铝板带箔等铝业项目，北方奔驰5万台重卡总装生产线、北方股份1000台非公路矿用车、包钢西创重型机械加工等装备制造项目进展顺利，有力地推动了传统产业的优化升级。积极培育战略性新兴产业，组织实施了79个重点项目。包钢高性能磁性材料、稀宝博为高端医用核磁共振、山晟光伏循环经济电池片组件、泛海一期60万吨甲醇等项目全面实施。特别是神华煤制烯烃一期生产进展顺利，包钢综合技改和兵总高端装备制造产业园区开工建设，对我市产业结构调整产生了重大的推动作用。实施“双百亿工程”成效显著，工业集群化水平进一步提高。新增北方重工、东方希铝2户百亿元企业，石拐工业园区、土右新型工业园区2个百亿元园区，全市百亿元企业、园区分别达到4个和6个，钢铁、装备制造业销售收入分别达到900亿元和800亿元以上。全面培育和发展中小企业，推动150户成长性高、创新性强、具有发展潜力的中小企业入选自治区千户成长型中小企业。全市规模以上工业完成增加值1237.3亿元，增长18.3%。科技创新能力不断增强，全社会研发经费达到29亿元，增长23%，自主研发的E260电动矿用车成功下线，稀土永磁器件用于“天宫一号”，AT变速器项目列入国家“863”计划，全市高新技术企业达到62户，占自治区的43%。

　　（二）服务业发展势头强劲。投资比重达到51.2%，增速达到31.8%。批零贸易、住宿餐饮、交通运输等传统服务业持续繁荣。包百、王府井、永盛成、万达广场、维多利等商贸企业不断做大做强，辐射带动能力进一步提升。市场体系日益完善，流通渠道更加畅通，城乡市场繁荣活跃。社会消费品零售总额完成847.5亿元，增长17.6%。物流、金融、旅游等现代服务业加快发展。确立了现代物流业“三基地、六园区、九中心”发展规划和思路，组织实施了土右煤炭物流、昆区钢铁化工物流、东河现代物流、石拐原材料物流和高新区润恒冷链物流等重点项目，一批档次高、活力强的物流企业进入我市。银行、保险、信托等金融业取得新成绩，年末金融机构本外币存款余额达到2001亿元，较年初增长16.8%；本外币贷款余额达到1287.9亿元，较年初增长22.9%。旅游业发展迈出新步伐，全年接待游客702.9万人次，旅游收入达到123.9亿元，分别增长16.5%和12.9%。会计、法律、信息、咨询、广告、中介等服务业得到进一步发展。

　　（三）城乡统筹取得积极进展。城乡一体化发展的基础不断夯实，四级规划体系编制工作基本完成，推进城乡一体化发展的若干意见制定出台，试点村建设成效明显。工业反哺农业、城市支持农村的力度逐步加大，全年用于“三农”方面的财政支出达到21.4亿元，增加6.1亿元。农业生产实现大丰收，达到历史最好水平。“南菜北薯、乳肉并举”战略深入实施。新增蔬菜保护地3万亩，新增滴灌、旱地覆膜马铃薯17万亩。优质肉羊良种率进一步提高，奶牛良种率达到100%。扎实推进农牧业产业化，规模以上加工企业实现销售收入71.9亿元，规模以上流通企业实现交易额202亿元。农田水利基础设施建设得到加强，新增有效灌溉面积3.26万亩、节水灌溉面积20.17万亩。全市耕种收综合机械化水平达到80.3%。扶贫开发工作取得新成绩，落实专项扶贫资金1.5亿元，实施各类扶贫项目145个，1.2万户、3.5万贫困人口直接受益。帮扶固阳县工作成效显著。全市牧区实现草原生态保护补助奖励政策全覆盖。石拐区被列入国家第三批资源枯竭型城市目录。

　　（四）城市建设力度进一步加大。立足包头的长远发展，进一步完善城市规划和设计。在全国范围内聘请9位知名专家担任顾问，为城市规划、设计、建设献计把脉。邀请国内外知名院所完成了钢铁大街与阿尔丁南大街、新都市区、南海湖地区、麻池与小白河地区等7个重点区域设计，对城区部分重要节点和在建的标志性建筑进行单体设计，为优化城市布局、规范空间开发秩序、提升城市品位奠定了基础。不断完善城市承载功能，全年实施城建重点项目158个，完成投资320亿元。一批市政基础设施项目先后完工，新增道路面积43万平方米，新增供热面积400万平方米，新建天然气加气站3座、管网56公里。一批群众关注的热点问题得到解决，治理旧小区48个、小街巷50条，完成200万平方米既有居住建筑节能改造，建成50个街道、社区服务中心，改善提高了43个居民小区的物业管理水平。积极组织实施绿化工程，植树37.2万株，新增绿地150万平方米，建成区绿化覆盖率达到41%。进一步强化城市管理，加大环卫设施投入力度，加强市政设施维护，整治违法建筑，清理户外广告，动员全社会扎实开展市容环境综合治理，城市面貌明显改善，荣获国家卫生城市的光荣称号。加快推进交通基础设施建设，集包第二双线工程、京藏高速公路包头段扩建、104省道百灵庙至白云鄂博、包头至石拐一级公路等工程顺利实施，211省道固阳至百灵庙段单幅通车，110国道土右旗段改造完成。

　　（五）环境治理扎实推进。全面实施“蓝天、绿水、青山、大草原”工程，生态建设和环境综合整治成效明显。积极推进“三片两线”环境综合治理。包钢下决心搬迁、淘汰落后产能，异地建设240万吨球团、210万吨焦化项目，淘汰两台90平方米烧结机，改造两台265平方米烧结机。集中治理整顿包钢周边121户企业，开工建设华业特钢异地搬迁项目，积极治理改造包头金属深加工园区。加强对稀土企业的整治和整合工作，依法关停13户稀土冶炼分离企业。取缔煤场和“十五小”违法排污企业319户，整治燃煤设施2011台。加强污水治理，加快污水处理厂、中水管网建设，全市污水日处理能力达到32.15万吨，生活污水处理率达到83%，再生水利用率达到35%。继续全力推进生态建设。完成各项林业建设任务57万亩，全市森林覆盖率达到15.7%，同比提高0.6个百分点。围封禁牧成果进一步巩固，山北草场植被平均盖度恢复到30%。水系建设加快实施，大青山生态应急水源和向山北地区输水工程主体接近完工。包头黄河国家湿地公园通过国家评审。

　　（六）改革开放不断深化。坚持以改革促发展，着力推进各项改革。企业改革进一步深化和完善，赛力特尔等6户企业改革收尾工作全部完成，华资电子等5户企业改制工作全面推开。启动了厂办大集体改革、解决国有企业职教幼教退休教师待遇问题等工作。按照国家的要求和部署，积极推进事业单位改革。深化医药卫生体制改革，实施基本药物制度成效明显。鼓励支持和引导非公有制经济加快发展。培育扶持劳动密集型、资源综合利用型、科技型中小企业，促进非公有制企业逐步向规模化、品牌化、外向型发展。全市私营企业突破2万户，增长14.6%，个体工商户突破9万户，增长28.7%。坚持以开放促发展，加大招商引资力度。组织参加了内蒙古·香港经贸合作活动周等大型招商活动，全年实施各类协作项目280项，引进国内（区外）资金实际到位370亿元，增长11.2%，全年利用外资12.4亿美元，增长12.7%。积极发展对外贸易，满都拉口岸和“无水港”过货量进一步增加，全市进出口总额完成27.7亿美元，增长41.8%。

　　（七）人民生活持续改善。各项惠民富民政策全面落实，财政用于民生方面的支出占到总支出的77%，为历史最高水平。全力做好就业工作，新增城镇就业4.08万人，其中已就业或落实就业去向的高校毕业生1.48万人，就业困难人员实现就业1.09万人，为6804名创业人员发放小额担保贷款4.49亿元，城镇登记失业率为3.87%。农村牧区劳动力转移就业22.4万人。进一步健全劳动关系协调三方机制，扩大工资集体协商范围，维护企业职工合法权益。努力提高社会保障水平，企业退休人员基本养老金月人均达到1743元，继续保持在自治区前列。稳步扩大社会保险覆盖面，“五七工”和老工伤人员分别纳入养老保险和工伤保险统筹。扩大新型农村牧区社会养老保险试点，参保人数达到33.6万人。新型农村牧区合作医疗实现市级统筹，参合率达到98.64%。关注困难群体，提高了城乡低保、五保户和城市“三无”人员供养标准，为12.7万人发放临时价格补贴，并建立了低收入居民生活补贴标准与物价上涨挂钩的联动机制。全年开工建设保障性住房8.91万套，为1.1万户低保家庭发放廉租住房补贴2000万元。城市健康水工程和农村牧区安全饮水工程加快推进，分别新增受益人口20.22万人和4.84万人。

　　（八）社会事业全面发展。健全公共文化服务体系，全市34个乡镇苏木综合文化站基本建成，旗县区“两馆”日臻完善。推出一系列文化惠民工程，保障了人民群众的基本文化需求。加强非物质文化遗产保护，“包头剪纸”和“梅日更召信俗”入选第三批国家级非物质文化遗产名录。推进创新型包头建设，科技工作取得新成效，连续七次荣获“全国科技进步先进市”称号。创新人才培养、选拔、引进和使用方式，推进人才资源整体开发，全市人才总量不断壮大，支撑作用得到强化。稀土高新区获评全国专利系统先进集体、全国知识产权试点示范工作先进集体、第三批国家级海外高层次人才创新创业基地。义务教育、高中教育、职业和成人教育取得新进展，高考成绩创近年来最好纪录。中小学校安工程、职教基地建设加快推进，改扩建幼儿园10所。群众性体育活动丰富多彩，竞技体育发展进一步加快。第十一届全国中学生运动会首次在非省会城市举办，我们克服场馆准备不足、经验缺乏等诸多困难，使中运会圆满成功，得到国家有关部委和各省市区领导的高度评价。大力发展卫生事业，完善公共卫生和医疗服务体系，新建4所县级医院病房楼、8所苏木乡镇卫生院和20个嘎查村卫生室，为社区卫生服务中心配备16台双向转诊服务车。人口和计生工作基本实现优质服务全覆盖。广播、电视综合覆盖率分别达到98.71%和98.65%，高于全国和自治区平均水平。妇女儿童、老年人、残疾人事业取得新成绩。人防、气象、地震、外事、史志、档案、仲裁等工作取得新进步。

　　（九）社会和谐稳定局面不断巩固。加强精神文明建设，广泛开展有地方特色的创建活动，完善长效机制，城市的整体文明程度不断提升，在激烈的竞争中继续蝉联全国文明城市荣誉称号。高度重视信访工作，畅通和规范群众诉求表达渠道，深入开展信访积案化解和领导干部接访活动，妥善解决了一批信访事项，维护了群众的合法权益。强化安全生产，认真开展重点领域专项整治，安全生产形势总体平稳。加强食品、药品监管，进一步规范和维护市场秩序。扎实推进“六五”普法工作。深入开展社会治安综合治理，严厉打击各类刑事犯罪。及时稳妥处置各种社会突发性事件，在自治区党委、政府的坚强领导下，妥善应对了锡盟两起刑事案件引发的群体性聚集事件，维护了社会稳定。惠龙集团非法吸收公众存款案件正在平稳处置。安排部署整治非法集资专项行动，有效防范和化解金融风险。全面落实党的民族宗教政策，各民族团结进步、繁荣发展的大好局面进一步巩固。积极支持国防动员和后备力量建设，实现全国双拥模范城“七连冠”和自治区双拥模范城“八连冠”。

　　各位代表，过去的一年，我们坚定不移地推进政府自身建设，认真执行市人大及其常委会的决议和决定，自觉接受市人大及其常委会的法律监督、工作监督和市政协的民主监督，加强同各民主党派、工商联和社会团体的联系。充分利用政府网站、新闻媒体、市长热线等平台，推进政务公开。依法清理政府规范性文件和行政执法机构，进一步规范行政审批程序，优化了政务服务环境。充分发挥行政监察、审计监督作用，严肃查处了一批违法违纪案件，维护了政府的良好形象。

　　总之，过去一年政府工作取得的成绩，凝聚着全体代表、各位委员的智慧和力量，是全市人民共同努力的结果。在此，我代表市政府，向全市各族干部群众，向人大代表、政协委员，向各民主党派、工商联、人民团体、社会各界，向中央和自治区驻包单位，向驻包解放军和武警官兵，向所有关心支持包头建设的同志们、朋友们，表示崇高的敬意和衷心的感谢！

　　在看到成绩和进步的同时，我们也清醒地认识到，发展中还存在不少困难和问题，主要是：综合实力不强，经济结构需要进一步调整优化；经济增长下行压力和物价上涨压力并存，稳增长、惠民生困难加大；资源环境约束日益突出，节能减排任务艰巨；城市管理仍有薄弱环节，为民便民水平需继续提高；高层次人才不足，科技创新能力有待加强；投资创业环境仍需改善，政务服务效能需要进一步提高；等等。对于这些问题，我们将高度重视，切实采取措施，认真加以解决。

　　二、2012年主要工作

　　2012年是全面贯彻市第十一次党代会精神的第一年，也是实施“十二五”规划承上启下的关键一年，我们将迎来党的十八大胜利召开。做好今年的工作，意义重大。

　　今年政府工作的总体思路和主要任务是：全面贯彻中央、自治区经济工作会议和市第十一次党代会精神，按照市委十一届二次全委（扩大）会议的部署和要求，牢牢把握科学发展主题和加快转变经济发展方式主线，贯彻“稳中求进”的总基调，大力实施“科学发展、全面转型、富民强市”战略，全力保持经济平稳较快发展，加快经济结构调整，加大城市建设和生态环境保护力度，深化改革扩大开放，切实保障和改善民生，全面推进各项社会事业，创新社会管理，维护和谐稳定，向提前实现全面建设小康社会目标迈出坚实步伐。

　　主要预期目标是：生产总值增长13.5%，财政收入增长17%，固定资产投资增长18%以上，城镇居民人均可支配收入和农牧民人均纯收入分别增长13%，城镇登记失业率控制在3.9%以内，人口自然增长率控制在5％。以内。完成自治区下达的节能降耗和主要污染物减排任务。确定这样的目标，综合了各方面因素，体现了中央和自治区突出强调稳增长的总体要求，虽然适当降低了经济增长预期，但对民生指标仍然保持了较高要求，两个收入目标衔接了“十二五”年均计划，力求实现两个同步，充分体现了富民优先的基本思路，符合实际，积极可行。

　　实现上述目标，要重点把握以下几个方面。一是把保持经济平稳较快发展作为首要任务。坚持稳中求进、以进促稳，准确把握宏观形势动向，有效破解发展难题，切实增强应对能力，用足用好国务院《关于进一步促进内蒙古经济社会又好又快发展的若干意见》、自治区支持呼包鄂地区发展等政策机遇，更加注重扩大投资和发展实体经济，抓好发展这个第一要务。二是把推动全面转型作为工作主线。坚持推进“五个转变”，深入实施“六项工程”，大力调整优化产业结构，全面开展生态宜居城市建设，扎实推进城乡一体化发展，继续抓好环境综合整治和生态建设，进一步强化科技和人才工作，以改革创新为根本动力，在发展中促转型、在转型中谋发展，努力开创经济、社会、生态全面转型发展的新局面。三是把保障和改善民生作为根本目的。坚持发展为了人民、发展依靠人民、发展的成果由人民共享，进一步加大民生工作力度，加强就业增收工作，提高社会保障水平，推进公共服务体系建设，创新社会管理，切实办好涉及民生的好事实事，在和谐稳定的社会环境中，提高发展的包容性和成果的普惠性，让人民群众生活得更加美好。

　　按照市委十一届二次全委（扩大）会议提出的“稳增长、促内需，调结构、促转型，抓城建、促统筹，治环境、促生态，惠民生、促和谐”等重点任务，市政府要狠抓工作落实，着重做好七个方面的工作：

　　（一）着力推动经济平稳较快发展。坚持实施重大项目带动战略，继续扩大投资规模，优化投资结构，增强投资拉动能力，采取有针对性的举措支持企业发展，促进经济持续健康增长。

　　拓宽筹资渠道。进一步加大招商引资力度，继续把招商引资作为扩大投资的主攻方向，充分发挥产业基础、生产成本等综合优势，主动承接沿海发达地区产业转移，加强对招商引资工作的策划和组织，积极引进一批新项目。创新招商引资方式，提高招商引资队伍专业化水平。充分发挥工商联、商会的优势，推进以商招商。积极组织参加内蒙古·香港经贸合作活动周等活动。全年力争引进国内（区外）资金400亿元以上。进一步扩大社会投资，发挥政府资金的引导作用，认真落实好国务院“新36条”，扩大民间资本进入领域，引导民间资本规范运行。积极争取国家和自治区资金，认真落实国家、自治区的支持政策，盯紧措施细化和项目建设，及时跟进争取。加强与国家、自治区有关部门的对接，扎实做好项目前期和申报工作，确保争取资金规模超过去年。充分发挥金融支持作用，积极协调金融机构增加信贷投放规模，增强对重大项目和实体经济的支持力度，确保新增银行贷款与经济发展同步增长。积极培育资本市场，创新融资方式，实现多元化投资、市场化运作。

　　加大投资力度。充分挖掘各行各业的投资需求，深入谋划扩大投资的重点方向、重点领域和重点项目。全年组织实施投资5000万元以上重点项目807个，年内完成投资1690亿元以上。加快产业项目建设，打造新的经济增长点。全年组织实施工业项目310个、商贸流通项目92个、农牧业项目42个，三次产业项目占到项目总数的55%。推进这些项目尽早开复工，加快建设进度，力争早日竣工释放生产能力。加大城乡建设力度，保持旺盛的投资需求。加快推进基础设施及能源交通、农田水利、生态环保、社会事业和改善民生等领域的项目建设。全面启动钢铁大街两侧原军分区、金融大厦、少年宫、图书馆、职工大学和赛力特尔等地块改造工程，加快重点区域改造开发，促进房地产业健康发展，保持投资对经济增长的拉动作用。

　　支持园区和企业发展壮大。进一步加大企业和园区的培育力度，充分发挥实体经济在稳增长中的基础作用。深入实施“双百亿工程”，积极推进企业和园区进一步做优做强，推动重点企业和园区向千亿级目标迈进。扶持和培育中小企业，促进集群化发展，形成集聚配套能力。优选200户技术含量及附加值高、有市场潜力的中小企业，在政策、资金、项目等方面给予倾斜。推进中小企业社会化服务体系建设，着力解决发展中的困难和问题，提升中小企业档次。坚持错位发展、体现产业特色，加快工业园区建设。抓好园区扩容升级，进一步优化园区发展环境，加大基础设施建设力度，完善管理体制机制，提高服务水平。开展园区“共建统管、税收分成”试点工作，完善园区建设目标考核体系，推动园区发展再上新台阶。努力把稀土高新区打造成全国一流创新型特色园区。

　　（二）着力促进产业结构转型升级。发挥老工业基地优势，坚持在增量中调整结构、在转型中优化升级，不断强化节能减排工作，构建多级支撑、结构优化的现代产业体系。

　　加快工业结构调整。深入落实自治区沿黄沿线产业带发展规划。推动钢铁、铝业、装备制造业等优势特色产业改造升级，力争钢铁产业和装备制造业产值均突破1000亿元。按照大型、高端、循环发展方向，加快实施包钢集团综合技改、包铝技改续建电解铝及自备电厂、希铝海平面镁合金循环经济等一批重点项目，打造以资源精深加工、产业上下游配套协作和循环发展为纽带的产业链。依托包头装备制造产业园区和一机集团、北方重工集团等大企业，加快推进兵总高端装备制造园建设，重点实施北创重载快捷铁路货车技改、北方装备巴里巴工程专用车、南京风电风机组装和齿轮箱制造、北京京城新能源兆瓦级风机生产等项目，推动装备制造业多元发展。在加强稀土资源保护的基础上，提高稀土应用水平，抓好包钢稀土冶炼分离集中加工、钕铁硼速凝薄带等重点项目建设。加快培育新能源、新材料、新化工等新型产业，积极发展节能环保、电子信息、新能源汽车、云计算等高新技术产业。重点推进山路集团扩建多晶硅单晶硅及太阳能电池片，国瑞碳谷碳纤维及复合材料，星和电机荧光粉、LED节能灯芯片，南京高精传动LED蓝宝石衬底等一批战略性新兴产业项目。争取启动神华煤化工二期工程，积极推动下游产业延伸加工。

　　提高服务业发展水平。大力发展现代物流业，突出打造“三基地、六园区、九中心”的物流产业发展格局，下大力气引进和建成几个规模大、档次高的物流园区，力争物流业增加值占第三产业的比重提高1个百分点。做大做强商贸流通业，完善现代商贸流通体系，突出抓好重点商务区项目，推进维多利、包商大厦等城市综合体建设。积极营造良好的消费环境，拓展消费服务领域和功能，培育消费热点，增强消费对经济的拉动作用。大力发展农村牧区服务业，深入实施“万村千乡市场工程”和“双百市场工程”，积极推进“农超对接”，不断完善农副产品市场流通体系。加快发展旅游业，抓好五当召、美岱召、沿黄湿地等旅游文化精品景区建设，加强旅游资源整合和宣传推介，促进旅游和文化产业融合发展。立足城市特色，大力发展工业旅游、民俗风情旅游，丰富形式和业态，提升旅游业档次。促进金融业健康发展，做大做强金融机构，创新金融服务，优化信贷结构，支持实体经济发展和保障性住房建设，提高金融风险应急管理和处置能力。扶持会展业，编制出台发展规划，确保会展中心投入使用。鼓励发展新型服务业，引导信息、研发、工业设计等生产性服务业和社区服务、养老服务等家庭服务业加快发展。

　　推动现代农牧业发展。继续深入实施“南菜北薯、乳肉并举”战略，提高农牧业规模化、设施化、标准化、产业化水平。加强菜篮子工程建设，全市蔬菜保护地面积力争新增4万亩。推进薯产业转型升级，加强优质马铃薯脱毒种薯繁育基地建设，全市马铃薯滴灌面积力争新增10万亩，主产区储藏水平达到50%以上。启动实施“奶业振兴、苜蓿发展”行动，加强标准化奶源基地建设，强化奶源监管，提高奶产业整体质量和效益。加快肉羊产业发展，按照数质并举和禁牧舍饲的要求，促进品种改良，扩大饲草生产规模。认真开展“农牧业科技促进年”活动，强化农牧业科技服务，完善农畜产品标准化生产、质量检测监管和重大动植物疫病防控体系，加大新品种、新技术、新设施等集成推广力度，提高农业耕种收综合机械化水平。

　　加大节能减排工作力度。强化对重点行业、重点领域、重点企业的节能减排目标管理，积极推进现有企业技术改造，严格项目准入，坚决淘汰落后产能和工艺。关闭淘汰、易地改造和就地治理一批污染企业，确保“三片两线”综合整治有较大突破。拆除包钢1—4号焦炉，加快华业特钢、明拓集团新厂区建设，实施大安、吉宇钢铁技术改造，鑫恒焦化转产改造等项目。加快污水收集和中水回用管网建设，推进东河东污水处理厂和北郊污水处理厂升级改造，加快西郊、万水泉和九原污水处理厂建设，年内污水日处理能力达到42万吨以上。把创建国家环保模范城市工作摆上重要议事日程，攻坚克难，深入实施创模重点工程。

　　（三）着力加快城乡建设步伐。坚持高起点规划、高标准建设、高强度投入、高水平管理，努力提高城市综合服务和承载功能，让包头变得更通畅、更绿色、更平安、更宜居，使群众生活得更加便利、舒心和幸福。

　　推进重点区域建设。完成重点区域的概念设计和延伸设计工作，提请市人大常委会对设计方案依法作出决议，逐步将城市设计成果付诸实施。加快钢铁大街中央商务区、新都市中心区建设，有序推进滨河新区、小白河地区、昆河沿岸和昆区南部区、东河北梁地区、石拐新区等重点区域建设。严格规范城市空间开发秩序，确保建一片成一片。实施城市景观绿化工程，加快赛汗塔拉城中湿地草原、友谊公园建设改造，推进劳动公园等5个公园、广场提档升级，改造民族东路、黄河大桥、东兴等城市出口周边环境，塑造城市新形象。

　　完善城市服务功能。针对热源不足、交通拥堵等问题，有针对性地加快市政基础设施建设，提升城市的便民水平和舒适度。新建2座热源厂，改造200万平方米老旧小区供热二次管网和10个小区的排水管网。完成200万平方米既有居住建筑节能改造。实施道路畅通工程，打通赛汗路、校园路等30条道路，渠化富强路与青年路交叉口等20处路口。加快“五横八纵”骨架路网建设，推进包钢西路等22条道路建设，实施钢铁大街、黄河大街等36条道路拓宽、绿化等综合改造，选择有条件的路段建设休闲绿道。继续抓好美化、亮化工程，亮化民族东路、阿尔丁南大街、文化路两侧建筑，规范设置城市出入口、火车站、飞机场等重要节点的店招牌匾。加快构筑城市现代交通体系，全面完成G6高速公路包头段扩建项目的征拆工作，加快实施110国道一级公路改建工程，积极推动省道211线、省道104线一级公路和包头至石拐一级公路建设，完善市域一级公路体系。加快推进城市轻轨项目和机场迁建前期工作。

　　切实加强城市管理。继续完善城市管理体制，促进城市管理精细化。加快停车场规划编制。制定地下管网规划和管理办法。健全完善长效机制，巩固创卫成果。优化公交线路，完善运营布局，加快公交车的更新配置。加大交通执法力度，努力使城市交通秩序进一步好转。加强对城乡结合部和拆迁改造区域的综合整治，坚决制止和依法打击私搭乱建等违法行为。加强土地收储工作，加快推进重点区域和重点地块土地储备和供应，做好经营城市这篇大文章。

　　加快城乡一体化进程。进一步完善城乡一体化发展的制度体系和配套政策。继续按照“规划先行、政策引导、重点突破、梯度推进”的要求，加快城乡统筹一体化发展步伐，把重点放在城中村、城边村改造上来，结合城市扩容、产业布局、环境整治、社会建设的新要求，加快推进19个城中村、城边村的改造。扎实推进新农村新牧区建设试点，重点在沿呼包银主轴线和山北交通干线有计划有选择地建设一批新农村示范村。加强县城和中心集镇规划建设，增强发展二三产业和集聚人口的功能。加快土右旗中等城市建设。加强城乡公共服务体系建设，为提升居民生活质量提供保障。实施向山北地区输水工程，确保年内向固阳供水，启动向达茂旗、白云矿区输水工程建设。改造和完善农村牧区供水管网，提高农村牧区自来水入户率、普及率。实施农村牧区安全饮水工程，解决4.5万农牧民饮水安全问题。加强农村牧区公路建设，建设通村公路175公里，新增25个通沥青（水泥）路的建制村，覆盖率达到60%以上。

　　优化生态环境。扎实推进林业生态建设工程，完成天然林资源保护、京津风沙源治理、“三北”防护林和退耕还林等国家重点工程建设任务30万亩以上，大青山南坡绿化二期工程建设任务1万亩。以56个村屯和黄河堤坝公路两侧绿化为重点，全力推进村屯绿化及通道绿化工程。加强大青山国家级自然保护区建设，依法打击私挖滥采行为，实施生态恢复治理工程，加快建设绿色生态屏障。继续巩固围封禁牧成果，加大禁牧督查和对农牧民生产生活支持的力度，认真落实草原生态补奖政策，确保禁牧工作不松劲、不反弹，实现草原生态体系持续恢复。严格执行黄河国家湿地公园规划，开展湿地环境综合整治和建设，进一步加强黄河湿地保护。推进大青山生态应急水源工程，加快建设南海湖等沿黄蓄滞洪区和昆河二期、四道沙河、二道沙河等河道综合整治工程。

　　（四）着力推进改革创新和对外开放。创新体制机制，实施更加积极的开放战略，为经济社会发展提供新动力、注入新活力。

　　坚持推进各项改革。继续做好国有企业改革工作，深化明天科技、华资实业和公交集团长途客运公司改革。加强国有资产监管，探索国有资本运营途径，不断提高国有资本运营质量。积极争取国家、自治区专项资金补贴，全力解决企业改制和历史遗留问题。做好厂办大集体改革、解决国有企业职教幼教退休教师待遇问题等工作。加快事业单位改革。深入推进文化体制改革。加强水资源管理和配置，深化水权转换改革。继续推进土地流转和规模经营，全年土地流转新增20万亩，达到170万亩以上。加快推进集体林权制度改革，全面完成确权发证工作。

　　不断扩大对外开放。积极开拓市场，鼓励企业扩大出口，保持出口平稳较快增长。加快“走出去”步伐，支持企业参与境外资源开发和经济合作。加大满都拉口岸基础设施建设力度，提高通关能力，扩大货运量，争取实现常年开放。推进国际集装箱转运站“无水港”建设。立足呼包鄂城市群发展，发挥区位优势，主动参与中西部分工协作，推进区域经济一体化。树立全方位开放理念，坚持“引资”和“引智”相结合，大力引进智力、人才和技术。举办好“中国包头稀土产业论坛”。

　　增强自主创新能力。以建设国家创新型城市为目标，充分发挥政府的引导作用，不断完善以企业为主体的创新体系。加大与知名院所的产学研合作力度，深化国内外科技合作交流。支持和培育高新技术企业和创新型企业发展，组织企业申报建设国家和自治区级研发机构。鼓励和引导企业增加投入，研发具有自主知识产权的核心技术和高附加值产品。全社会研发经费占生产总值比重提高到1.1%。加快推进清华科技园、北师大文化创意园、中科院包头稀土研究所等项目建设，争取批复博士工作站。组织实施自治区“草原英才”工程、呼包鄂“草原硅谷”建设方案、“5512”专业技术人才工程和“青年创新人才培养计划”，为科技创新提供人才保障。

　　发展壮大非公有制经济。认真贯彻落实国家关于支持非公有制经济发展的政策，完善配套措施，健全创业辅导、融资担保、技术支持服务体系，创造公平竞争、平等准入的市场环境。帮助和支持有条件的民营企业上市融资、发行债券。加快中小企业创业园区建设，扶持民营企业围绕优势特色产业搞延伸、围绕重点项目搞协作，进一步引导民营企业向实体经济发展。

　　（五）着力保障和改善民生。怀着对人民群众的深厚感情和满腔热情，把民生工作进一步做实、做细、做好。继续加大财政投入力度，把资金花在群众最需要的地方，让群众得到更多实惠。集中力量为人民群众兴办12件好事实事。

　　千方百计扩大就业。扩大小额担保贷款合作金融机构，建立创业园区或创业孵化基地，积极促进创业带动就业。制定就业服务与重点项目对接的政策措施，最大限度发挥大项目拉动就业的作用。通过鼓励企业吸纳、公益性岗位安置、扶持自主创业等措施，多渠道促进城镇低收入家庭失业人员就业。加大就业培训力度，提高城乡劳动者的技能水平。全年城镇新增就业4万人，其中城镇下岗失业人员再就业2万人。

　　继续增加城乡居民收入。落实好增加群众收入的政策措施，提高最低工资标准，完善企业职工工资集体协商机制，促进职工收入与企业效益同步增长。增加居民财产性收入。努力拓宽农牧民增收渠道，认真落实相关补贴政策，继续强化劳务输出，促进农牧民产业增收、政策增收和务工增收。全年农村牧区劳动力转移就业达到23万人。把扶贫开发作为“头号民生工程”来抓，以增收为核心，落实好国家、自治区扶贫政策，完善专项扶贫、行业扶贫和社会扶贫模式，全力打好新一轮扶贫开发攻坚战。支持帮扶困难地区加快发展，继续开展帮扶固阳县工作。

　　进一步提升社会保障水平。完善各类从业人员养老、医疗、工伤、失业和生育保险制度，扩大城乡居民养老保险和城镇居民医疗保险覆盖面。把全额拨款事业单位纳入工伤保险统筹，实现工伤保险全覆盖。继续提高企业退休人员养老金标准。开展城镇居民医疗保险门诊统筹，提高基本医疗保险政策内报销比例。提高新型农村牧区合作医疗人均筹资标准和报销比例，参合率保持在98%以上。为重点优抚对象每人每月增加20元抚恤和定补费。提高城乡最低生活保障标准和五保户供养标准，发放低收入老人高龄津贴。为环卫一线工人提高月工资水平200元。加强对城乡低保对象、低保边缘人员、低收入人员等困难群体的救助、帮扶工作，完善低收入居民生活补贴标准与物价上涨挂钩的联动机制。继续实施“健康水”工程，新增受益群众10万人。大力推进保障性安居工程建设，新增廉租住房货币补贴1500户，新开工保障性住房30892套，帮助更多低收入群体解决住房困难。

　　努力维护人民群众切身利益。进一步健全安全生产管理体制和机制，全面落实安全生产责任。加强对交通、矿山、化工等重点行业的安全监管，做好消防安全工作，强化应急救援机制建设。深入开展专项整治，认真排查各种隐患，确保不发生重特大事故。加强社会治安综合治理，严厉打击各类违法犯罪，积极推进全社会视频监控报警联网系统建设，深入开展平安创建活动。认真落实稳控物价各项政策，切实加强市场物价监管，保持物价基本稳定。强化粮食安全意识，组织实施放心粮油工程。进一步强化食品、药品安全监管，全力抓好专项治理，构建长效机制，完善食品、药品安全保障体系，让放心食品、药品走进千家万户，让人民群众吃得放心、用得安心。

　　（六）着力推进社会建设和管理创新。立足于促进社会和谐，推进社会管理和创新，大力发展各项社会事业，增加公共产品供给，更好地满足人民群众需求。

　　加强社会管理和创新。积极稳妥地开展“精街道、强社区”试点工作，加强基层基础建设，整合基层社会管理资源，构建综合管理服务平台，充实强化社区管理服务力量，全面提升社区管理和服务水平。构建信访、调解、综治“三位一体”的社会矛盾排查化解机制，努力把矛盾化解在基层和萌芽状态。坚持以群众工作统揽信访工作全局，扎实开展“治理整顿信访秩序年”活动，下功夫抓好信访工作，确保信访态势稳定回落。加快社会诚信体系建设，建立健全覆盖全社会的征信系统。全面贯彻党的民族宗教政策，广泛开展民族团结进步宣传和创建活动。加强国防教育，重视边防建设，完善国防动员体制，加强民兵和预备役部队建设，做好拥军优属、拥政爱民工作。

　　坚持优先发展教育。加快发展学前教育，新建、改扩建30所公立幼儿园，有效缓解适龄幼儿“入园难”问题。推进义务教育均衡发展，改造40所农村牧区义务教育薄弱学校，重点解决农村牧区义务教育学校大班额、学生住宿困难、教学设备短缺等问题。完善义务教育经费保障机制，加快落实高中阶段免费教育。提高教师素质和教学质量。强化校车安全。全面完成校安工程三年规划任务。继续推进素质教育，促进学生德智体美全面发展。高度重视民族教育，继续做好蒙中、回中搬迁工作。加快职教基地建设进程，提升职业教育办学水平，完成6所职院搬迁工作。支持高等教育发展，为包头发展提供人才支撑。

　　加快建设文化大市。按照构建“三个体系”、推进“三个专项工作”、强化“四项保障措施”的总体要求，促进文化事业全面繁荣和文化产业快速发展。把社会主义核心价值体系融入国民教育和精神文明建设全过程，加强职业道德、社会公德、家庭美德和个人品德建设，广泛开展群众性精神文明建设活动，巩固全国文明城市创建成果，不断提升公民文明素质和城乡文明程度。培养文化艺术人才，繁荣文艺创作，弘扬主旋律，不断推出精品力作。健全公共文化服务体系，加快包头大剧院等重点文化项目建设，完善旗县区“两馆”功能，提高公共文化服务水平。继续开展文化惠民活动，推进草原书屋等工程，加快广播电视数字化进程。积极创建全国公共文化服务体系示范区。进一步挖掘历史文化资源和城市特色文化，筹建文化产业园区，发展出版发行、影视制作、演艺娱乐和动漫等文化产业。继续做好社会科学、文物保护、图书档案等工作。

　　提高人民群众健康水平。优化卫生医疗资源配置，加大标准化社区卫生服务机构建设力度，提高社区卫生服务保障能力。加强基层医疗卫生设施建设，完成1所县级医院、4所苏木乡镇卫生院、20所嘎查村卫生室建设，加快推进18个社区卫生服务中心建设。加大农村牧区医疗卫生队伍培养力度。加强医疗服务管理，努力为群众提供安全、有效、方便、价廉的医疗服务，着力解决看病贵、就医难的问题。完善公共卫生服务体系，抓好重大疾病防治，有效应对突发公共卫生事件。做好人口和计划生育工作，提高出生人口素质。大力开展群众性体育活动，提高竞技运动水平。继续做好妇女儿童、老年人和残疾人工作。

　　（七）着力加强政府自身建设。以建设法治政府、服务政府、责任政府和效能政府为目标，提高推动科学发展的能力和水平。

　　坚持依法行政。坚决贯彻落实市委决策，自觉接受人大及其常委会的法律监督、工作监督和政协的民主监督，认真听取各民主党派、工商联、无党派人士和各人民团体的意见。加强政府系统内部监督，支持监察、审计部门依法独立履行监督职责。加强行政执法监督，建立健全责任追究制和绩效考评制，使各项工作责任有主体、实施有监督、结果有考核。大力推进政务公开和事务公开，增强政府工作透明度，保障人民群众的知情权和参与权，推进政府工作的规范化、民主化、法制化。

　　坚持高效理政。深入推进政府职能转变，加强政府效能建设，把政府工作的着力点放到谋大事、抓重点、解决突出问题上。坚定不移地推进行政审批制度改革，减少和规范行政审批事项。完善“一站式办公”、“投资绿色通道”等审批形式，年内力争建成政务服务中心并投入使用。优化财政支出结构、强化预算管理，提高财政资金使用效益。加快电子政务建设，进一步推行网上办公和网上办事，提高政务信息化水平。

　　坚持务实勤政。坚持实事求是，一切从实际出发、一切从群众利益出发，多做打基础、利长远、得民心、顺民意的工作。坚持深入群众调查研究，摸实情、办实事、求实效。继续办好“市长热线”和“政风行风热线”。大力倡导立说立行、埋头苦干、一抓到底的工作作风，增强服务发展、服务基层、服务群众的能力。

　　坚持从严治政。加强党风廉政建设，全面落实“一岗双责”，加大源头治腐力度，完善惩治和预防腐败体系。加强机关作风建设，继续开展民主评议政风行风活动，坚决纠正部门和行业不正之风，切实解决损害人民群众利益的突出问题。树立过紧日子的思想，严格控制“三公”经费支出。加强对公务员的教育、管理和监督，努力建设一支政治强、业务精、作风好的高素质公务员队伍。

　　各位代表，让我们在自治区党委、政府和市委的领导下，深入贯彻落实科学发展观，大力实施“科学发展、全面转型、富民强市”战略，振奋精神，团结奋进，开拓创新，以优异成绩迎接党的十八大胜利召开！
